# Gambling, suicide and mental health treatment utilisation in Wales: case–control, whole-population-based study

**DOI:** 10.1192/bjo.2025.10867

**Published:** 2025-10-07

**Authors:** Matthew Jones, Pippa Boering, Kishan Patel, Daniel Leightley, Simon Dymond

**Affiliations:** School of Psychology, Swansea University, Swansea, UK; Gambling Harm UK, London, UK; Department of Population Health Sciences, School of Life Course & Population Sciences, King’s College London, London, UK; Department of Psychology, Reykjavík University, Reykjavík, Iceland

**Keywords:** Gambling, suicide, routinely collected data, health records, mental health treatment

## Abstract

**Background:**

Gambling-related harm is a global public health concern. Suicide mortality is increased among people who experience gambling harm, and people who die by suicide often have contact with mental health treatment services in the months preceding their death.

**Aims:**

To assess via a case–control study how gambling diagnosis predicts suicidal death and mental healthcare utilisation using linked routinely collected healthcare data.

**Method:**

We linked the Welsh Longitudinal General Practice Dataset, Annual District Death Extract, Patient Episode Database for Wales, and Outpatient Appointments Dataset Wales using the Secure Anonymised Information Linkage (SAIL) Databank. A sample of individuals with gambling diagnosis who died by suicide and an age- and sex-matched comparator group of all-cause decedents between 1993 and 2023 were extracted. Predictors of suicidal death, including mental health diagnosis and treatment contacts, were analysed using binary logistic regression models and chi-squared tests.

**Results:**

A matched cohort of 92 individuals diagnosed with a gambling diagnosis (mean age 61.5 years, s.d. 13.1; 71% male) who died by suicide and 2990 comparators were identified. Gambling diagnosis status was a significant predictor of suicide (odds ratio 30.94; 95% CI 3.57–268.28; *P* = 0.002). Individuals with gambling disorder had significantly more mental health treatment contacts (*P* < 0.001), particularly in-patient contacts (*P* < 0.001). No difference in out-patient contacts was found.

**Conclusions:**

Historical diagnosis of gambling harm is a significant predictor of suicidal death and mental health treatment utilisation. Improved screening and coding practices would facilitate greater data linkage research on gambling-related suicide and suicide prevention.

Gambling has become ubiquitous, with almost half (46.2%; 95% CI 41.7–50.8) of adults globally reporting gambling in the past year.^1^ Gambling harm caused by problems related to gambling is a major public health challenge.^2^ Problematic patterns of gambling, which are those that cause multiple problems for the individual in personal, family, financial and employment domains, are estimated to have a global prevalence of 1.4% (95% CI 1.06–1.84).^1^ A further 8.7% (95% CI 6.6–11.3) of adults engage in ‘any risk’ gambling, which includes individuals who experience at least one adverse consequence from gambling.^1^ Persistent or recurrent patterns of gambling form part of the diagnostic criteria for gambling disorder within both the DSM-5 and the ICD-11. Globally, up to 2.2% (0.9–3.9) of adults experience problematic gambling or gambling disorder; this includes up to 54.7 (23.403–99.583) million men and 25.3 (11.483–44.607) million women. As these estimates illustrate, the scale and extent of gambling harm show no signs of abating, and neither do the increasing costs of clinical treatment and support services.

Gambling disorder is associated with increased risks of suicide, mortality and comorbidity.^3–6^ In a meta-analytic literature review, Kristensen et al^4^ identified pooled prevalence rates of 31.6% (95% CI 29.1–34.3) for lifetime suicidal ideation and 13.2% (95% CI 11.3–15.5) for lifetime suicide attempts, respectively, among individuals with gambling problems. To date, however, few studies have sought to investigate the association between gambling disorder and suicide mortality.^7^ Using a whole-population cohort design with routinely collected data from Norway, Kristensen et al^8^ found that suicide was the leading cause of death among individuals with gambling disorder between 2008 and 2021. That is, among 6899 people with gambling disorder, 37 of 148 deaths (25%) were by suicide. Moreover, suicide risk did not differ from that seen with people with comorbid conditions such as anxiety disorders, depression and personality disorders. These findings indicate that gambling disorder is associated with increased suicide risk in a potentially separable manner from that involving comorbid disorders.^3^ Other studies linked patient registries in Sweden and Canada, respectively, with national cause of death data sources and found increased risk of suicide among individuals recorded as ‘gambling’ cases.^3,8,9^ To infer gambling caseness, two studies analysed the records of individuals with a diagnosis of ‘gambling disorder’ defined as ‘pathological gambling’ (ICD-10: F63.0) using Read codes from ICD-10, whereas another defined gambling as ‘a history of gambling’.^9^ Previous linked registry-based studies^3,8^ have found suicide to be the leading cause of mortality among people with a gambling disorder diagnosis and have demonstrated increased suicide mortality rates (SMRs) for people with a diagnosis compared with the general population in Sweden (SMR 5.1; 95% CI 3.71–7.06) and Norway (SMR 15.1; 95% CI 8.7–21.6), respectively. Reccord et al^9^ assessed differences in suicide rates between rural and urban areas in Canada and found a significant association between gambling and suicide (*P* = 0.007). To date, these are the only existing data linkage studies of gambling-related suicide using routinely collected data.^7^

Healthcare contacts recorded through routinely collected data present opportunities for early intervention and treatment, as well as potential data sources for further research.^10^ Routinely collected data – that is, data gathered from administrative and clinical records – may provide both increased statistical power owing to large sample sizes and insight into the pragmatic nature of healthcare contact and treatment.^11^ Linkage of a range of data-sets from services such as general practice, out-patient appointments, hospital admissions and annual deaths can provide a comprehensive understanding of how individuals experiencing gambling harm access healthcare before their death. Individuals who die by suicide are more likely to access health services, including general practice and emergency departments, in the year before their death.^12,13^ Evidence from Wales (in the UK) has shown that 98.3% of those who died by suicide had been in contact with healthcare services in the year before their death, and healthcare contacts were higher in the week before their death. Individuals who died by suicide were also more likely to attend emergency departments rather than primary care or out-patient services.^14^

Globally, help-seeking rates for gambling are low, and many people experiencing harm from gambling never seek treatment.^15^ In the UK, individuals experiencing problematic gambling may be more likely to consult their general practitioner for mental health concerns and to be admitted to hospital and receive psychological counselling compared with those who do not gamble.^16^ A Canadian study found that individuals with a history of problem gambling who subsequently died by suicide had lower rates of accessing front-line health and social services in the year leading up to their death and throughout their lives.^17^ High rates of comorbidity with mental disorders including depression, schizophrenia, generalised anxiety disorder, obsessive–compulsive disorder, panic disorder and personality disorders, as well as addictive behaviours such as alcohol use disorder, increase the range of potential mental health treatment-seeking opportunities experienced by people with gambling disorder.

To date, no study has examined the association between gambling diagnoses and mental health treatment utilisation in the months preceding death by suicide. Here, we conducted a study of healthcare contacts, gambling, suicide and mental health disorders using population-based linked data at the person level, with a case–control design, across a study period of 30 years. Our primary aim was to assess for the first time the degree to which gambling predicts suicidal death using routinely collected healthcare data. Our secondary aim was to measure the degree to which gambling diagnoses predict mental health treatment utilisation.

## Method

### Study design

**Fig. 1:**
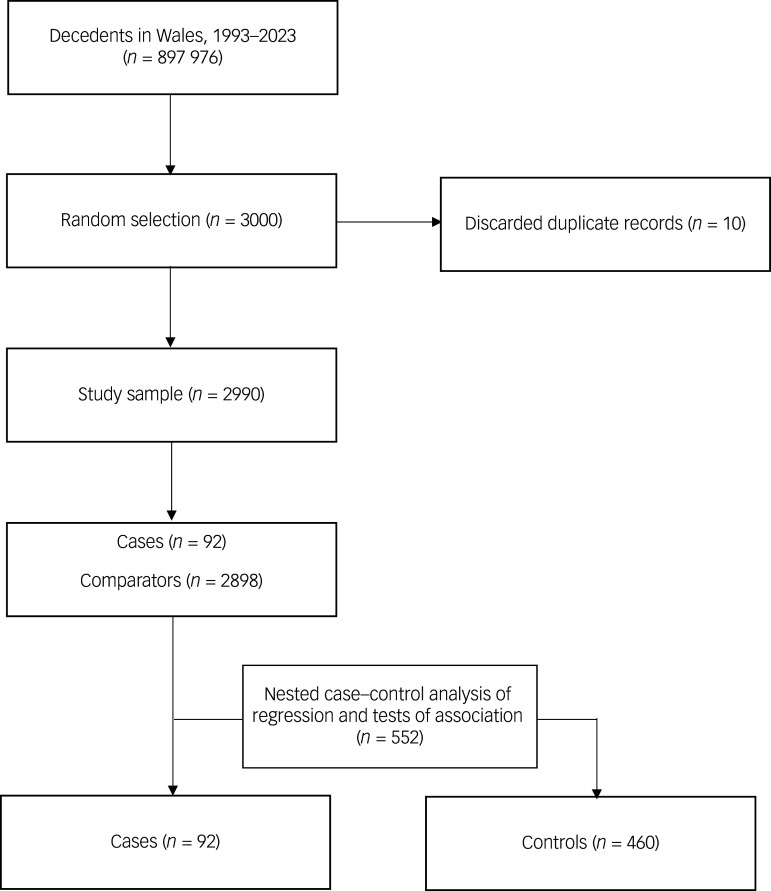
Flow diagram of cases and controls.

We conducted a case–control study to compare individuals who died by suicide and had a past diagnosis of gambling in Wales between 1 January 1993 and 31 December 2023 with those who did not die by suicide (Fig. 1). A random sample of 3000 all-cause decedents were selected as comparators (using the SQL ‘rand’ function to identify a random sample). This comparator sample size was deemed optimal for clinical prediction models.^18–20^ Gambling was defined with either pathological/compulsive gambling or betting (primary care data Read codes E2C31, Xa2ke, Eu63000-1, ZV4k500) and gambling and betting or pathological gambling (hospital admission ICD-10 codes Z72.6 and F63.0; Table 1). Suicidal death was defined as fatal intentional self-harm in accordance with the Office for National Statistics (ICD-10 codes X60-X84; see Supplementary Materials available at https://doi.org/10.1192/bjo.2025.10867).^21–23^

**Table 1:** Inclusion criteria for cases

Coding framework	Diagnostic code	Description	Male	Female
Read coding (general practice events)	E2C31 Xa2ke Eu63000 Eu63011 ZV4k500	Pathological gambling Compulsive gambling Pathological gambling Pathological gambling Gambling and betting	*n* = 33 (35.87%) *Φ* *Φ* *Φ* *n* = 18 (20.65%)	*n* = 14 (15.22%) *Φ* *Φ* *Φ* *Φ*
ICD-10 coding (e.g. in-patient and out-patient contacts)	Z72.6 F63.0	Gambling and betting Pathological gambling	*n* = 15 (16.3%) *Φ*	*Φ* *Φ*

Φ is the count suppressed due to low cell size following statistical reporting requirements.

### Data sources

Gambling diagnoses, suicide deaths and health contacts were obtained from the Welsh Longitudinal General Practice Dataset, the Annual District Death Extract), the Patient Episode Database for Wales, and the Outpatient Dataset for Wales (Table 2), all housed within the Secure Anonymised Information Linkage (SAIL) Databank.^20,24^ SAIL is a privacy-protecting Trusted Research Environment holding anonymised data from the whole population of Wales. We undertook probabilistic linkage of health and mortality records using a unique identifier (anonymised linkage field) generated by SAIL.

**Table 2:** Included data-sets linked in SAIL, variables and coding framework

Data-set	Variables	Coding framework/methodology
Welsh Longitudinal General Practice Dataset	Clinical events, diagnostic coding	Read coded thesaurus of clinical terms versions 2 and 3
Annual District Death Extract	Underlying cause of death, date of death, age at death, sex	ICD-10
Patient Episode Database for Wales	In-patient stays and day-case appointments, diagnostic coding, in-patient length of stay	ICD-10
Outpatient Appointments Dataset Wales	Out-patient appointments, diagnostic coding	ICD-10

### Case and comparator definitions

We defined gambling cases as those with either primary care data Read codes or hospital admission ICD-10 codes for gambling, with death recorded as suicide and an index date of death between 1993 and 2023. Within SAIL, we age- and sex-matched each gambling case to five all-cause decedents who had also had contact with primary care, emergency departments, hospitals and out-patient services in Wales since 1993.

### Measures and variables

#### Demographics

We extracted sex and age at death from the Annual District Death Extract data source (Table 2).

#### Diagnostic variables

We extracted mental health diagnostic variables assigned for cases and comparators based on disorders known to be highly comorbid with gambling, such as alcohol use disorder and depression,^25,26^ and other disorders including generalised anxiety disorder, obsessive–compulsive disorder, personality disorders, schizophrenia, schizotypal and delusional disorders, post-traumatic stress disorder and panic disorder. Further details of the coding frameworks and data sources can be found in the Supplementary Material.

#### Healthcare contacts

A healthcare ‘contact’ was a recorded entry in healthcare records. Contacts included those recorded in primary care (e.g. general practice settings), hospital admissions with an episode with a psychiatric specialty or treatment (Read coded thesaurus of clinical terms versions 2 and 3), mental health primary diagnosis (ICD-10 codes ‘F’), and out-patient attendances with a psychiatric specialty or treatment.

### Statistical methods

Individual record linkage and generation of data tables were conducted using Eclipse software version 2024-12 R for Windows (Eclipse Foundation, Belgium; https://eclipseide.org/) and SQL within SAIL. Descriptive statistics were produced before matching of cases to comparators.^27^ Binary logistic regressions with predictor variables of any treatment contact related to gambling diagnosis (e.g. caseness), mental health diagnoses in in-patient (including day-cases), out-patient and primary care (i.e. general practitioner) settings were conducted. We conducted nested case–control analysis of regression and tests of association for cases (*n* = 92) and controls (*n* = 460) (Fig. 1). The backward elimination method of stepped regression was used to assess predictors of suicide, and all predictors which did not contribute to the model and exhibited high *P*-values were removed. The Hosmer–Lemeshow test was used to determine goodness of fit. Chi-squared tests of association were conducted, with mental health treatment contacts as the dependent variable (*n* = 552) and gambling, alcohol and mental health diagnoses as independent variables. The eta coefficient was used as an effect size estimate.

### Ethics statement

This study makes use of patient data held in the SAIL Databank; individuals’ informed consent was not necessary, as all data were anonymised. The authors assert that all procedures contributing to this work comply with the ethical standards of the relevant national and institutional committees, and with the Helsinki Declaration of 1975, as revised in 2013. Ethical approval was obtained from SAIL’s Information Governance Review Panel (project number 1564).

## Results

### Study population

We identified a cohort of *n* = 92 gambling cases who died by suicide. After ten duplicate records had been removed from the all-cause decedents (*n* = 897 976), we identified a final group of *n* = 2990 comparators.

Individuals with a history of gambling disorder who died by suicide were 61.5 years old on average (s.d. 13.1), and the majority were male (71%). Almost half of cases had a depression diagnosis (45.65%) and 25% had an alcohol-related diagnosis. Most gambling diagnoses were made in the general practice setting (51.09%), and 26.09% were made in an in-patient setting (Table 3). Compared with the control group, individuals in the case cohort were younger (61.5 years (s.d. 13.1) for cases *v*. 79.56 years (s.d. 12.35) for controls) and had a higher prevalence of depression and alcohol use disorder the year before death (Table 3).

**Table 3:** Demographic characteristics, mental health (depression), alcohol use and gambling diagnoses in primary and secondary care settings and suicidal deaths among cases and comparators in the year before death

Variable	Cases (*n* = 92)	Comparators (*n* = 2990)	*z*-statistic	*P*
Mean age, years (s.d.)	61.52 (13.14)	79.56 (12.35)	–	–
Gender	71% male, 29% female	72% male, 28% female	–	–
Depression diagnosis (out-patient and in-patient including day-cases), *n* (%)	42 (45.65)	542 (18.13)	–	–
Alcohol-related diagnosis (out-patient and in-patient including day-cases), *n* (%)	23 (25)	177 (5.9)	–	–
In-hospital (in-patient and daycare) gambling-related diagnosis, *n* (%)	24 (26.09)	–	–	–
General practice gambling-related diagnosis, *n* (%)	47 (51.09)	–	–	–
Suicidal death	≤10%	≤1%	8.65	<0.001

Most gambling-related diagnostic codes (47%) were found in primary care data rather than in-hospital data (24%). Crucially, cases and comparators differed significantly with respect to the incidence of suicidal death only.

### Mental health predictors of suicidal death

Having a history of gambling disorder was a predictor of suicidal death (odds ratio 30.94 (95% CI 3.57–268.28), *P* = 0.002). In this model, alcohol use disorders, depression, and schizophrenia, schizotypal and delusional disorders were not significant predictors of suicide (Table 4). The Hosmer–Lemeshow test returned a greater *P*-value, indicating improved goodness of fit (*χ* ^2^ = 1.48, d.f. = 8, *P* = 0.83), and the omnibus test was significant at *χ* ^2^ = 22.16, d.f. = 4 and *P* ≤ 0.001.

**Table 4:** Mental health predictors of suicidal death (*n* = 552)

Diagnostic predictor	Odds ratio	s.e.	*P*	95% CI
Gambling	30.94	1.1	0.002	3.57–268.28
Alcohol	0.79	0.917	0.81	0.13–4.82
Depression	0.78	0.79	0.76	0.17–3.7
Schizophrenia, schizotypal and delusional disorders	3.86	0.83	0.1	0.76–19.52

### Mental health treatment contact

Mental health treatment utilisation is summarised in Table 5. We found that cases had more frequent contact with mental health treatment services overall (*P* < 0.001) than comparators, particularly in-patient contacts (*P* < 0.001); there was no significant difference for out-patient contacts.^a^ As shown in Table 6, only individuals with a gambling diagnosis were significantly likely to have had mental health treatment contacts before their death (*P* = 0.005).

**Table 5:** Mental health treatment utilisation (contact) characteristics

Variable	Cases (*n* = 92) *χ* ^2^ (s.d.)	Comparators (*n* = 2990) *χ* ^2^ (s.d.)	*t*-statistic	*P*
Mean mental health treatment contacts (out-patient and in-patient including day-case appointments)	3.41 (2.87)	1.81 (2.87)	3.52	<0.001
Out-patient mental health treatment contacts	0.58 (1.72)	0.78 (2.11)	−1.09	−0.2
In-patient mental health treatment contacts (including day-case appointments)	2.83 (4.08)	1.04 (1.81)	4.19	<0.001

**Table 6:** Chi-squared tests of association (*n* = 552) between mental health treatment contacts

Variable	*χ* ^2^	d.f.	*P*	*η*
Gambling	14.96	4	0.005	0.15
Alcohol	2.58	4	0.63	0.008
Depression	2.05	4	0.72	0.01
Schizophrenia, schizotypal and delusional disorders	7.67	4	0.1	0.03
Generalised anxiety disorder	0.11	4	0.98	0.01
Obsessive–compulsive disorder	0.07	4	0.99	0.009
Panic disorder	4.6	4	0.33	0.008
Personality disorders	0.28	4	0.99	0.17

## Discussion

We found that those with a gambling diagnosis who died by suicide (cases) during a 30-year period in Wales were more likely to be male, to have comorbid depression and to have received their diagnosis in general practice settings. Cases were significantly more likely to have died by suicide compared with those who died by other means (comparators) and had more frequent contact with mental health treatment services before their death. These findings are the first to confirm the predictive relationship between gambling and suicide using routinely collected healthcare data via population-level cohort designs with linked UK data-sets. As such, the present study adds to the growing global evidence base on gambling-related suicide using such designs.^3,8,28^ Our findings also extend research on contacts with primary and secondary healthcare^14^ to mental health in-patient treatment contacts for gambling-related harm and subsequent suicide.

Significantly more deaths by suicide were seen among cases than among comparators. The suicide rate in 2023 in Wales, with a population of 3.1 million, was 14 deaths per 100 000.^29,30^ We found that suicidal death was recorded for fewer than 10% of cases (suppressed owing to low cell size); allowing for rounding, this was still more than ten times greater than the rate in the general population.^29^ Despite this, the proportion of cases with death due to suicide was substantially lower than that reported by a recent Nordic study of registry data, which found that a quarter of all deaths of people with a diagnosis of gambling disorder between 2008 and 2021 were by suicide.^8^ In that study, people with a gambling disorder diagnosis were at greater risk of suicide than patients with other psychiatric, neurological and cardiovascular conditions. Similarly, we found that suicide was not associated with prior conditions such as alcohol use disorder, depression, or schizophrenia, schizotypal and delusional disorders.^25,26^ This effect persisted despite cases having a greater prevalence of depression and alcohol use disorders the year before death. Thus, the risk of suicide among people with gambling disorder seems to be independent of the onset of prior comorbid conditions.^3^

We found that individuals with a history of gambling who died by suicide were more likely to have had more frequent contact with mental health treatment services before their death. This was in line with the findings of Kristensen et al,^8^ who noted that people with gambling disorder who died by suicide had more contact with specialist healthcare services for intentional self-harm (X60–X84) than those with a gambling disorder diagnosis who had not died by suicide. A history of or current self-harm is a known risk factor for suicide among psychiatric in-patients,^31^ people recently discharged^32^ and those contacting secondary healthcare settings.^33,34^ Mental health treatment services form part of suicide prevention services in those presenting with self-harm^35^ and offer an opportunity to intervene, most notably in terms of repeat contacts with healthcare services and/or clinical or Read coding for gambling. Our findings indicate that a history of gambling may be a contributory factor in the help-seeking of those who die by suicide and emphasise the importance of accurately screening for gambling-related harm among those who have contact with primary or secondary healthcare services for self-harm.

### Strengths and limitations

The present study is the first to use routinely collected healthcare data from the UK to identify predictors of suicidal death among people with historical gambling diagnoses.^7^ Data were of adequate quality, and our data linkage methods were robust. Given that both suicide and gambling disorder are relatively rare outcomes, the present use of linked routinely collected data enabled a larger sample size, conferred greater statistical power than observational methods and facilitated generalisability. However, this study may have had limitations. Routinely collected data are contingent on medical coding practices in various healthcare settings and may be affected by misclassification bias, particularly when dealing with potentially stigmatising outcomes such as gambling disorder. Therefore, it is key that future research should focus on both improving routine data coding and underdiagnosis of gambling harm.

Coding of gambling diagnoses in general practice and hospital settings was relatively infrequent; considering the observation period, the size of the sample, and the estimated population prevalence of gambling related harms,^1^ it is possible that our sample was not representative of the true number of decedents in Wales with a history of gambling diagnosis. The comparator group was significantly older than the case group, which may have affected healthcare utilisation.^36^ We could not draw conclusions about the type of gambling or the medium by which gambling activities were accessed (e.g. online or land-based). Globally, the risk of problematic gambling is greatest among those who engage in online gambling,^1,2,37^ and only one in five people experiencing problematic gambling seek help for the harm caused by their gambling.^15^ It is likely, therefore, that the number of cases we identified was an underestimate of the extent of help-seeking and healthcare utilisation among people harmed by online gambling and other forms of gambling.

Although we attempted to match participants by age, cases were older on average than the comparison group. Depending on the characteristics of the study population, this can be a common limitation in observational research, and caution in interpreting age-related findings is advised. Given that age is necessarily predictive of morbidity and mortality, residual confounding of our outcomes cannot be ruled out. However, our findings regarding caseness and age at death were robust in terms of strength of association and significance.

The presence of a gambling-related diagnosis being the only significant mental health predictor of both suicidal death and mental health treatment utilisation may have been due, at least in part, to the limited number of severe cases and the low incidence of suicide in our control group, which may have disproportionately increased the predictive power of caseness. Those with severe presentations of other mental health disorders may be less likely to contact health services.^34,38^ Finally, our use of death date as the index date, although consistent with previous studies,^14,34^ may have overlooked earlier opportunities for healthcare services to intervene.

### Implications for policy and practice

Contrasting how prior gambling diagnosis predicts suicidal death and healthcare utilisation using linked routinely collected healthcare data affords early intervention opportunities for suicide prevention. Screening for gambling-related harm should be conducted in primary and secondary healthcare settings where possible, and resources devoted to staff training and raising awareness of help and support.^39,40^ Future research should also explore barriers and obstacles to wider adoption of clinical coding among healthcare professionals and ensuring the integrity and completeness of routinely collected data in research on gambling harm.^7^

## Supporting information

Jones et al. supplementary material Jones et al. supplementary material

## Data Availability

Access to SAIL data is available on application to the SAIL Databank via their usage governance process (www.saildatabank.com).

## Footnotes

a. As mental health treatment contacts occurred before and following gambling diagnoses, the temporal relationship between caseness and mental health treatment utilisation was not suitable for regression.

## Supplementary material

The supplementary material is available online at https://doi.org/10.1192/bjo.2025.10867
