# Supplementary material for: Gambling, suicide and mental health treatment utilisation in Wales: case–control, whole-population-based study
Source: BJPsych Open. 2025 Oct 7;11(6):e234. doi: 10.1192/bjo.2025.10867 (PMC12529343; doi:10.1192/bjo.2025.10867)
Supplement: Jones et al. supplementary material [file S2056472425108673sup001.docx]

**Supplementary Material**

*Table S1.* Underlying cause of death mapped to ICD-10 code.

| **Coding framework** | **Diagnostic codes** | **Description** |
| --- | --- | --- |
| International Classification of Diseases version 10 (ICD-10) | X60   X61    X62   X63   X64   X65  X66   X67  X68  X69   X70  X71  X72 X73  X74  X75 X76 X77  X78 X79 X80  X81  X82  X83 X84 | Intentional self-poisoning by and exposure to nonopioid analgesics, antipyretics and antirheumatics Intentional self-poisoning by and exposure to antiepileptic, sedative-hypnotic, antiparkinsonism and psychotropic drugs, not elsewhere classified Intentional self-poisoning by and exposure to narcotics and psychodysleptics [hallucinogens], not elsewhere classified Intentional self-poisoning by and exposure to other drugs acting on the autonomic nervous system Intentional self-poisoning by and exposure to other and unspecified drugs, medicaments and biological substances Intentional self-poisoning by and exposure to alcohol Intentional self-poisoning by and exposure to organic solvents and halogenated hydrocarbons and their vapours Intentional self-poisoning by and exposure to carbon monoxide and other gases and vapours Intentional self-poisoning by and exposure to pesticides Intentional self-poisoning by and exposure to other and unspecified chemicals and noxious substances Intentional self-harm by hanging, strangulation and suffocation Intentional self-harm by drowning and submersion Intentional self-harm by handgun discharge Intentional self-harm by rifle, shotgun and larger firearm discharge Intentional self-harm by other and unspecified firearm discharge Intentional self-harm by explosive material Intentional self-harm by smoke, fire and flames Intentional self-harm by steam, hot vapours and hot objects Intentional self-harm by sharp object Intentional self-harm by blunt object Intentional self-harm by jumping from a high place Intentional self-harm by jumping or lying before moving object Intentional self-harm by crashing of motor vehicle Intentional self-harm by other specified means Intentional self-harm by unspecified means |

*Table S2.* Clinical Read and ICD-10 codes used to identify other variables of interest*.*

| **Coding framework** | **Diagnostic codes** | **Description** |
| --- | --- | --- |
| Read coded thesaurus of clinical terms versions 2 and 3 (CTV2, CTV3) | XaB9J XaCIs 1465. XSEGJ XaCIu E2B1. X00Sb XE1Zb XaCIt E2112 Xa1yZ  Xa2lt  XE1YX  E2500  E2501  E2502  E2503  E250z  E2002  E203.  X764L  E2030  E2031  E203z  Eu42 Eu420  Eu42y  Eu421  Eu422  Eu42z  X00SW  E2024  E2023  E2025  X761k  Xa0Xc  Xa0xd  XE1Y7 Eu410  E2021 E2022  XE2b6  E2152  XE1YF  E215.  E21y0  E2019  E21y4  E21y3  E2111  E21y.  E21y1  E21y5  E21y6  X00S6 Eu20.  E2122  XE1ZO  Eu25.  E1..  X00Qy  E103.  E101.  E102.  E10y0  E10..  E10y1  E10z  Eu20z  XE1Xx  XSGon  X00Sf  XaX58  XaX56  XaX55  E2831  E29y1  XaEFB | Depression NOS  Mild depression  History of depression  Major depression  Severe depression  Chronic depression  Mixed anxiety and depression  Depressive episode, unspecified  Moderate depression  Dysthymia  Alcohol abuse  Persistent alcohol abuse  Nondependent alcohol abuse  Nondependent alcohol abuse, unspecified  Nondependent alcohol abuse, continuous  Nondependent alcohol abuse, episodic  Nondependent alcohol abuse, in remission  Nondependent alcohol abuse, NOS Generalised anxiety disorder  Obsessive-compulsive disorders  Obsessive-compulsive behaviour  Compulsive neurosis  Obsessional neurosis  Obsessive-compulsive disorder NOS  Obsessive-compulsive disorder  Predominantly obsessional thoughts or ruminations  Other obsessive-compulsive disorders  Predominantly compulsive acts [obsessional rituals]  Mixed obsessional thoughts and acts  Obsessive-compulsive disorder, unspecified  Social phobia Social phobia, fear of public speaking Social phobia, fear of public eating  Social phobia, fear of public washing Anxiety about social functioning, behaviour or performance Anxiety about appearing ridiculous Anxiety about saying the wrong thing Panic disorder  Panic disorder [episodic paroxysmal anxiety]  Agoraphobia with panic attacks  Agoraphobia without mention of panic attacks Personality disorder  Emotionally unstable personality disorder  Paranoid personality disorder  Hysterical personality disorder  Narcissistic personality disorder  Dissociative personality disorder  Eccentric personality disorder  Passive aggressive personality disorder  Hypomanic personality disorder  Other personality disorder  Avoidant personality disorder  Immature personality disorder  Masochistic personality disorder  Psychotic disorder  Paranoid schizophrenia  Schizotypal personality disorder  Delusional disorder  Schizoaffective disorder  Non-organic psychoses  Reactive psychosis  Paranoid schizophrenia  Hebephrenic schizophrenia  Catatonic schizophrenia  Other schizophrenia  Schizophrenic disorders  Cenesthopathic schizophrenia  Schizophrenia NOS  Schizophrenia, unspecified  Other schizophrenia  Severe major depression with psychotic features  Post-traumatic stress disorder  Delayed post-traumatic stress disorder following military combat  Chronic post-traumatic stress disorder following military combat  Acute post-traumatic stress disorder following military combat  Acute post-trauma stress state  Other post-traumatic stress disorder  Chronic post-traumatic stress disorder |
| International Classification of Diseases version 10 (ICD-10) | F32.0  F32.1  F32.2  F32.3  F32.8  F32.9  F10.0  F10.1  F10.2  F10.3  F10.4 F10.5  F10.6  F10.7  F10.8  F10.9  F41.1  F41.2  F41.3  F41.8  F41.9  F42  F42.0  F42.1  F42.2  F42.8  F42.9  F40.1  F41.0  F60.0  F60.1  F60.2  F60.3  F60.4 F60.5  F60.6 F60.7 F60.8 F60.9  F20.0 F20.1 F20.2 F20.3 F20.4 F20.5 F20.6 F20.8 F20.9 F21 F22.0  F22.8  F22.9  F23.0  F23.1  F23.2  F23.3  F23.8  F23.9  F24 F25.0  F25.1  F25.2  F25.8 F25.9  F28  F29  F43.1 F43.0 | Mild depressive episode  Moderate depressive episode  Severe depressive episode without psychotic symptoms  Severe depressive episode with psychotic symptoms  Other depressive episodes  Depressive episodes, unspecified  Acute alcohol intoxication  Harmful use of alcohol  Alcohol dependence  Alcohol withdrawal  Alcohol withdrawal delirium  Alcohol psychosis  Alcohol amnesia Residual alcohol psychosis Other alcohol use disorder  Unspecified alcohol use disorder  Generalised anxiety disorder  Mixed anxiety and depression  Other mixed anxiety  Other specified anxiety  Anxiety disorder unspecified  Obsessive-compulsive disorder  Predominant obsessions  Predominant compulsions  Mixed obsessions and compulsions  Other obsessive-compulsive disorders Obsessive-compulsive disorder, unspecified Social anxiety disorder Panic disorder  Paranoid personality disorder Schizoid personality disorder Dissocial personality disorder Emotionally unstable personality disorder Histrionic personality disorder Anankastic personality disorder Avoidant personality disorder Dependent personality disorder Other personality disorder Personality disorder, unspecified Paranoid schizophrenia Hebephrenic schizophrenia Catatonic schizophrenia Undifferentiated schizophrenia Post-schizophrenic depression Residual schizophrenia Simple schizophrenia Other schizophrenia Schizophrenia, unspecified Schizotypal disorder Delusional disorder Other persistent delusional disorder Persistent delusional disorder, unspecified Acute polymorphic psychotic disorder without symptoms of schizophrenia Acute polymorphic psychotic disorder with symptoms of schizophrenia Acute schizophrenia-like psychotic disorder Other acute predominantly delusional psychotic disorders Other acute and transient psychotic disorders Acute and transient psychotic disorder, unspecified Induced delusional disorder Schizoaffective disorder, manic type Schizoaffective disorder, depressive type Schizoaffective disorder, mixed type Other schizoaffective disorders Schizoaffective disorder, unspecified Other nonorganic psychotic disorders Unspecified nonorganic psychosis PTSD Acute stress reaction |
|  |  |  |

*Table S3.* Mental health predictors of suicidal death (n=552).

| **Diagnostic predictor** | **OR** | **Std. error** | **p** | **95% CI** | |
| --- | --- | --- | --- | --- | --- |
| Gambling | 32.25 | 1.1 | 0.002 | 3.71 | 280.22 |
| Alcohol | 0.97 | 0.91 | 0.97 | 0.16 | 5.78 |
| Depression | 0.98 | 0.79 | 0.98 | 0.21 | 4.65 |
| GAD | 0. | 15036.3 | 0.99 | 0. | 0. |
| OCD | 0. | 18326.65 | 0.99 | 0. | 0. |
| Panic disorder | 0. | 4733.78 | 0.99 | 0. | 0. |
| PTSD | 0. | 14687.76 | 0.99 | 0. | 0. |
| Schizophrenia, schizotypal and delusional disorders | 3.61 | 0.83 | 0.12 | 0.71 | 18.31 |
| Personality disorders | 0. | 9300.251 | 0.99 | 0. | 0. |
